# Is it worth paying attention to actinedid mites in agricultural fields?

**DOI:** 10.1007/s10493-024-00981-w

**Published:** 2025-01-16

**Authors:** Veronika Gergócs-Winkler, Norbert Flórián, Zsolt Tóth, László Sipőcz, Miklós Dombos

**Affiliations:** Institute for Soil Sciences, HUN-REN Centre for Agricultural Research, Fehérvári út 144, Budapest, H- 1116 Hungary

**Keywords:** Endeostigmata, Grass, Heterostigmata, Maize, Prostigmata, Wheat, Seasonality

## Abstract

Agricultural management increases the seasonal dynamics of soil-dwelling organisms compared to natural habitats. Our knowledge is very poor about the relationship between seasonal changes of soil microorganisms and the microbivorous soil arthropods. To reveal these connections, we have to know more about the seasonal changes of soil-dwelling microarthropods in croplands. Actinedid mites are rarely the subject of synecological studies, however, this group regularly reaches the dominant part of mite assemblages in agro-ecosystems. In this study, we investigated the seasonal density changes of actinedid mites from two independent studies of agricultural fields. Soil samples were taken from maize and wheat fields for two years, and from newly established meadows for one year in summer and autumn in Hungary. Soil-dwelling mites were enumerated and identified at the suborder level and soil parameters were measured. Actinedid mites dominated most of our soil samples. The density of Endeostigmata was the highest in the summer and the density of Heterostigmata was the highest in the autumn within one year among different crop species, soil types, and years. Endeostigmatid mites had negative relationships with soil nitrogen parameters and positive with soil moisture. Heterostigmatid mites had various relationships with soil moisture. The ecology of actinedid mites is under-examined but their high number in agricultural fields may justify the fact that they should receive more attention. We assume that Actinedida, mainly Endeostigmata and Heterostigmata are worth to investigate in croplands as a starting point to reveal the connection between the seasonality of soil mites and soil microbiota.

**Supplementary Information:**

The online version contains supplementary material available at 10.1007/s10493-024-00981-w.

## Introduction

Agricultural management seriously influences soil ecological processes (Sanaullah et al. 2020). Besides climatic parameters, fertilisation, tillage, harvest, pesticide- and herbicide usage have high effects on temporal changes of soil organisms (Lauber et al. 2013; Maurício Rumenos Guidetti Zagatto et al. 2017). Therefore, in agro-ecosystems, seasonal dynamics of soil microorganisms are more pronounced than in natural habitats (Lauber et al. 2013; Chernov and Zhelezova 2020). There is much evidence about what influences seasonal changes of soil microbiota in crop lands, which include soil organic matter (Pereira e Silva et al. 2012), plant development (Kaiser and Heinemeyer 1993; Ryan et al. 2009), climatic and soil parameters (Guo and Siepel 2020). The only poorly studied factor is the soil-dwelling microbivores and fungivores (Chernov and Zhelezova 2020).

In agro-ecosystems, the most abundant microbivorous and fungivorous soil fauna includes mites (Acari) and springtails (Collembola) (Kumar et al. 2020), and in some cases, the most abundant ones of them are actinedid mites. Actinedida is the old name of a paraphyletic mite group including the suborder Prostigmata (order: Trombidiformes) and the suborder Endeostigmata (order: Sarcoptiformes) (Krantz and Walter 2009; Russell et al. 2010). Several studies revealed that Actinedida can reach more than 70% of the mite abundance in different agricultural fields (Emmanuel et al. 1985; Kethley 1990; Osler et al. 2008; Van Leeuwen et al. 2015).

Prostigmata is a very diverse group according to morphology and ecology having predaceous, phytophagous, fungivorous, parasitic, and parasitoid species with a wide range of body sizes (300−12,000 μm) and habitat preferences (Krantz and Walter 2009). Within the diverse Prostigmata suborder, we distinguish the morphologically less diverse cohort of Heterostigmatina (Heterostigmata) having mainly fungivorous species in croplands (Gormsen et al. 2006). Communities of heterostigmatid mites can be influenced by abiotic parameters such as soil moisture (Eitminavičiūtė et al. 2008; Beyer et al. 2011) and by biotic parameters such as soil organic matter (Russell et al. 2010) and soil microbial assemblages (Emmanuel et al. 1985; Cao et al. 2011). Endeostigmata consists mainly of small-sized (200–300 μm), microphytophagous or detritivore species (Walter 1988). Most of the studies indicates that endeostigmatid mites are influenced by soil moisture or precipitation (Steinberger and Ben-Ythak 1990; Kinnear and Tongway 2004; Rieff et al. 2014) and by soil organic matter (Russell et al. 2010).

Studies about seasonal changes of Actinedida in agricultural fields are very poor. Maybe because not all the agricultural fields are dominated by them (Gruss et al. 2013; Twardowski et al. 2017). However, croplands dominated by microbivorous and fungivorous Actinedida groups may reflect considerable patterns related to soil microorganisms. Therefore, we find it important to investigate the temporal patterns of these mites in agro-ecosystems.

In two of our previous studies, we experienced high absolute and relative abundance and repeating seasonal patterns of these mites in different croplands. The main goal of the recent study is to draw attention to the significance of these poorly-known, soil-dwelling mites in agricultural fields. The high density of actinedid mites may indicate their important role in soil ecological processes. Our first hypothesis is that these mites are the most abundant mite group in all the studied crop fields. We also want to show that these mites have repeating temporal (seasonal) patterns in agricultural fields. The second hypothesis is that the seasonal patterns of Actinedid mites occur in all the studied fields. Finally, we also hypothesised that there are soil parameters which can be related to the abundance changes of actinedid mites.

## Materials and methods

### Description of the study sites

#### Study #1: wheat and maize fields

**Fig. 1:**
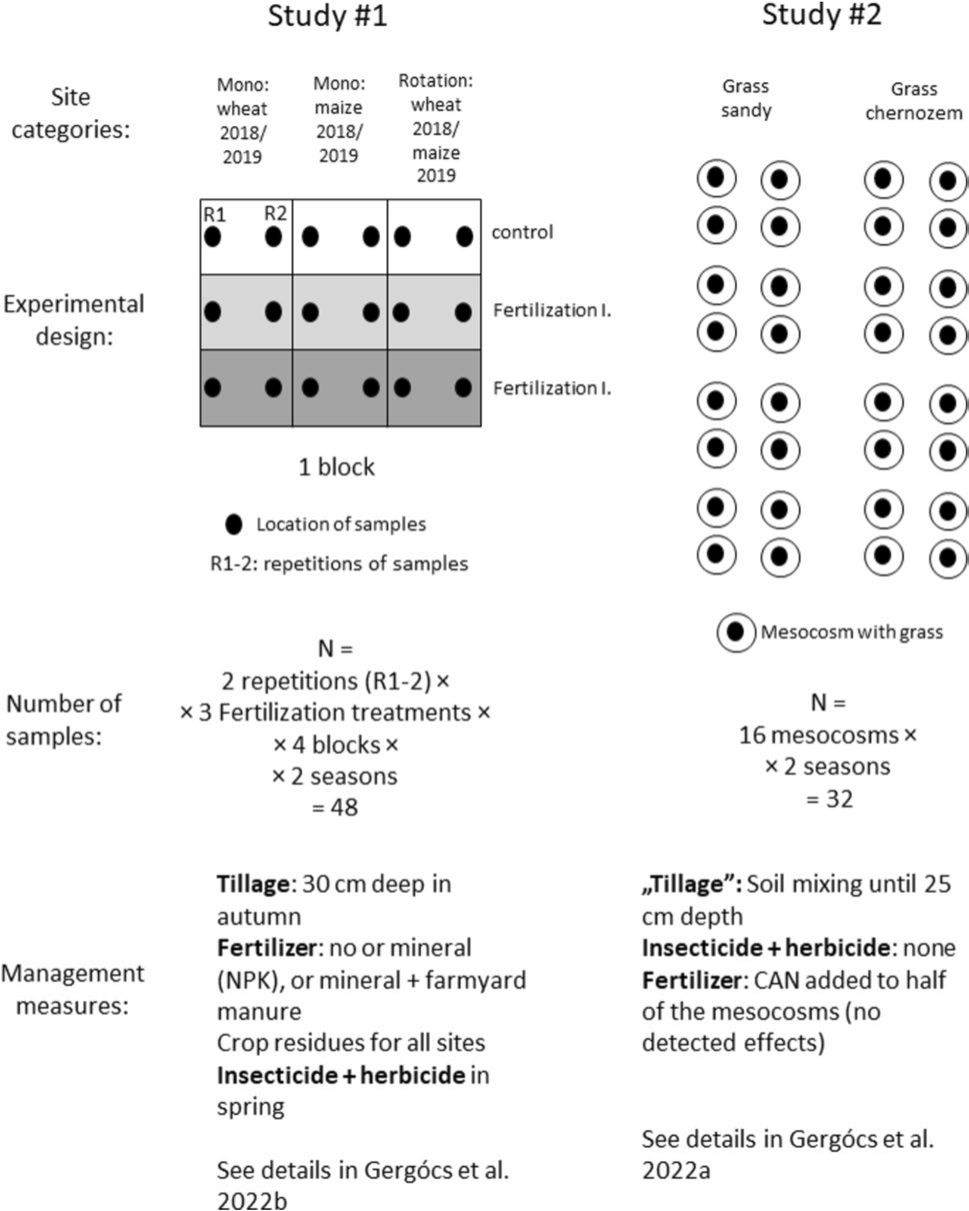
Schematic experimental design of the two studies

The first study was a two-year (2018–2019) investigation of a long-term fertilization experiment in wheat *(Triticum aestivum L.)* and maize *(Zea mays L.)* fields in Martonvásár, Hungary (47°19′53.25′′ N, 18°47′22.06′′ E) (for details see (Gergócs et al. 2022a). The soil type was Chernic Phaeozem (WRB: Loamic, Aric). The study used three types of cropping systems (monoculture wheat, monoculture maize and a wheat-maize crop rotation, ‘biculture’ (2018: wheat, 2019: maize) and three types of fertilization practices (no fertilization, mineral fertilization and mineral + organic fertilization) giving nine study plots in four blocks (Fig. 1). In each study plot (33 × 7 m), two undisturbed soil samples (400 cm^3^, 8 cm in depth) were taken in June and October 2018 and 2019. The samples of different times were taken within a 1 m^2^ area. Thus, a study plot has two sampling points (R1 and R2). Six site categories were considered: monoculture wheat 2018 and 2019; monoculture maize 2018 and 2019, finally, biculture wheat 2018 and biculture maize 2019. Each site category originally had 48 samples (48 = 2 repetitions × 3 fertilization types × 4 blocks × 2 seasons) (Fig. 1). These samples were extracted in 70% ethyl-alcohol with Berlese-funnel for seven days and mites were counted. We separated the two years because seasonality was in focus and biculture sites were incomparable due to the different crop plants in the two years.

Soil properties [(NO_2_ + NO_3_)-N concentration (mg kg^−1^), NH_4_–N content (mg kg^−1^) and humus (m%, g g^−1^), pH, P_2_O_5_ ion concentration (mg kg^−1^)] were determined by the Szolnok Soil Protection Lab. Ltd according to Hungarian standards (Gergócs et al. 2022b. Soil moisture was determined using the thermo-gravimetric method (m%, g g^−1^) drying samples for 24 h at 105 °C. Climatic data about the preceding periods before sampling are in Table 1.

#### Study #2: freshly planted grass

In the second study, the seasonal abundance changes of soil-dwelling mites were investigated in freshly planted grass in 2021. The study was conducted in two small, half-year-old grass plots (previously a wheat field) within agricultural landscapes, in two types of soil in Hungary [calcareous sandy soil (WRB: Mollic Umbrisol, Arenic)] in Őrbottyán (47°40′10.15″N, 19°15′12.15″E) and calcareous chernozem soil (WRB: Calcaric Phaeozem) in Nagyhörcsök (46°51′56.62″N, 18°31′08.41″E)]. In each grass plot, 16 pieces of PVC cylinder tubes were inserted in the ground (diameter: 40 cm, depth: 20 cm) and filled back with soil (Fig. 1). Visible plant particles were removed from the soil by hand. On 19th March, a mixture of four perennial grass species was sown in the tubes (40% *Festuca rubra*, L., 20% *Festuca heterophylla*, Lam., 20% *Lolium perenne*, L., and 20% *Festuca arundinacea*, Schreb.). Grass within the tubes was irrigated preventing desiccation and was cut (at the height of 5 cm) at the two sampling times. From each tube, one soil core sample was taken (400 cm^3^, 8 cm in depth) on 6th July and 12th October in the same way as in the previous study. Here, the two site categories were “grass sandy” and “grass chernozem” with 32 samples from the 16 mesocosms and two seasons. Microarthropods were extracted from the soil core with Berlese-funnel for seven days in 70% ethyl-alcohol and then, mites were counted. Details about the study sites see in Gergócs et al. (2022b).

Soil NH_4_–N (mg kg^−1^) and NO_3_–N contents (mg kg^−1^) of the soil were determined according to steam distillation methods (Bremner and Keeney 1965; Bremner 2016). Soil humus content (m%, g g^−1^) was determined by the Hungarian standard (Gergócs et al. 2022b). Soil moisture (V% cm^3 ^cm^−3^) was determined with a Campbell CS616 Water Content Reflectometer. Climatic data about the preceding periods before sampling are in Table 1.

**Table 1:** Climate data of the study sites during the studies

	Study #1				Study #2			
Study site	Martonvásár				Nagyhörcsök		Őrbottyán	
Soil	Chernozem				Chernozem		Sandy	
Plant	Wheat and maize				Grass		Grass	
Year	2018	2018	2019	2019	2021	2021	2021	2021
Season	Summer	Autumn	Summer	Autumn	Summer	Autumn	Summer	Autumn
Temp. (°C)	18	14.3	13.4	16.1	24.1	15.8	22.4	14.4
Prec. (mm)	62.7	2.6	138.6	11.4	81.7*	34.3*	89.3*	26.3*

Temperature values are averages of daily mean temperatures for the month preceding the sampling

Precipitation values are the sum of precipitation values for the month preceding the sampling

*Precipitation and irrigation combined

### Mite groups

In all studies, mite specimens (adults and immatures) were enumerated and determined at different levels of groups (Oribatida, Mesostigmata, Astigmata, Prostigmata) (Krantz and Walter 2009) with stereo-microscope (15–45× magnification). For the separation of Prostigmata into Endeostigmata, Heterostigmata and “other Prostigmata”, at the beginning of the determination work, some specimens of the different morphospecies in each group were treated with lactic acid, and determined with light microscope (up to 200× magnification) using the keys of Krantz and Walter (2009).

### Statistical analyses

Density data (individuals m^−2^) of mite groups (Oribatida, Mesostigmata, Astigmata, Prostigmata, Heterostigmata, Endeostigmata) were considered in the eight site categories and their mean density data were displayed in two stacked bar charts performed with R Studio (R version 4.2.1, (R Core Team 2019) with the ggplot function (ggplot2 package (Wickham 2011) for summer (June or July) and autumn (October or November).

Density data (ind. m^−2^) of the three mite groups (Endeostigmata, Heterostigmata, and Prostigmata) were demonstrated on seasonally clustered bar plots performed with the ggplot function. N values (data points) were indicated for the whole site category, not separating the seasons. In some categories, N values may differ from the original N values because some samples were lost.

Mite density values were compared between the two seasons in linear regression models or generalised least square models (factors: seasons and site categories) for the three mite groups separately with the functions lm() or gls() (package: nlme, Pinheiro et al. 2024). Mite density was log transformed before analyses. According to lowest AIC values, generalised least square models with factor determined variance structure were used in most of the cases [varIdent(form = 1 | factor(season) × factor(site)] to correct heterogeneity of model residual variance. Significant differences between the seasons within a site category were calculated with function emmeans() and contrast() (package emmeans, Lenth 2024).

Relative density changes of the three mite groups were calculated between the two seasons for each sampling point in each site category as follows:

$$\user2{Formula~}1\user2{~~~~~~}~relative~density~change = ~\frac{{autumn~density - summer~density}}{{summer~density}}$$

When the summer density was zero, this value was replaced by the number one. These relative density values were demonstrated in box plots for each site category performed in R Studio.

The relation between mite densities and soil variables were analysed with linear regression models (lm) and generalised least square (gls) models (package: nlme). For study #1, the two repeated samples (R1 and R2) from the same study plot were averaged for mite densities because the soil parameters were present only for each study plot. If heterogeneity of residuals occurred in linear regression models generalised least square models with the adequate variance structure were used. The variance structure was selected according to the lowest AIC values. The significant fixed term of the models was selected with maximum likelihood estimation using function anova() (package: stats). The models were performed separately for each plant species. In addition, the soil variables had very different values between the two soil types in study #2, so the two soil types were also separately analysed.

## Results

**Fig. 2:**
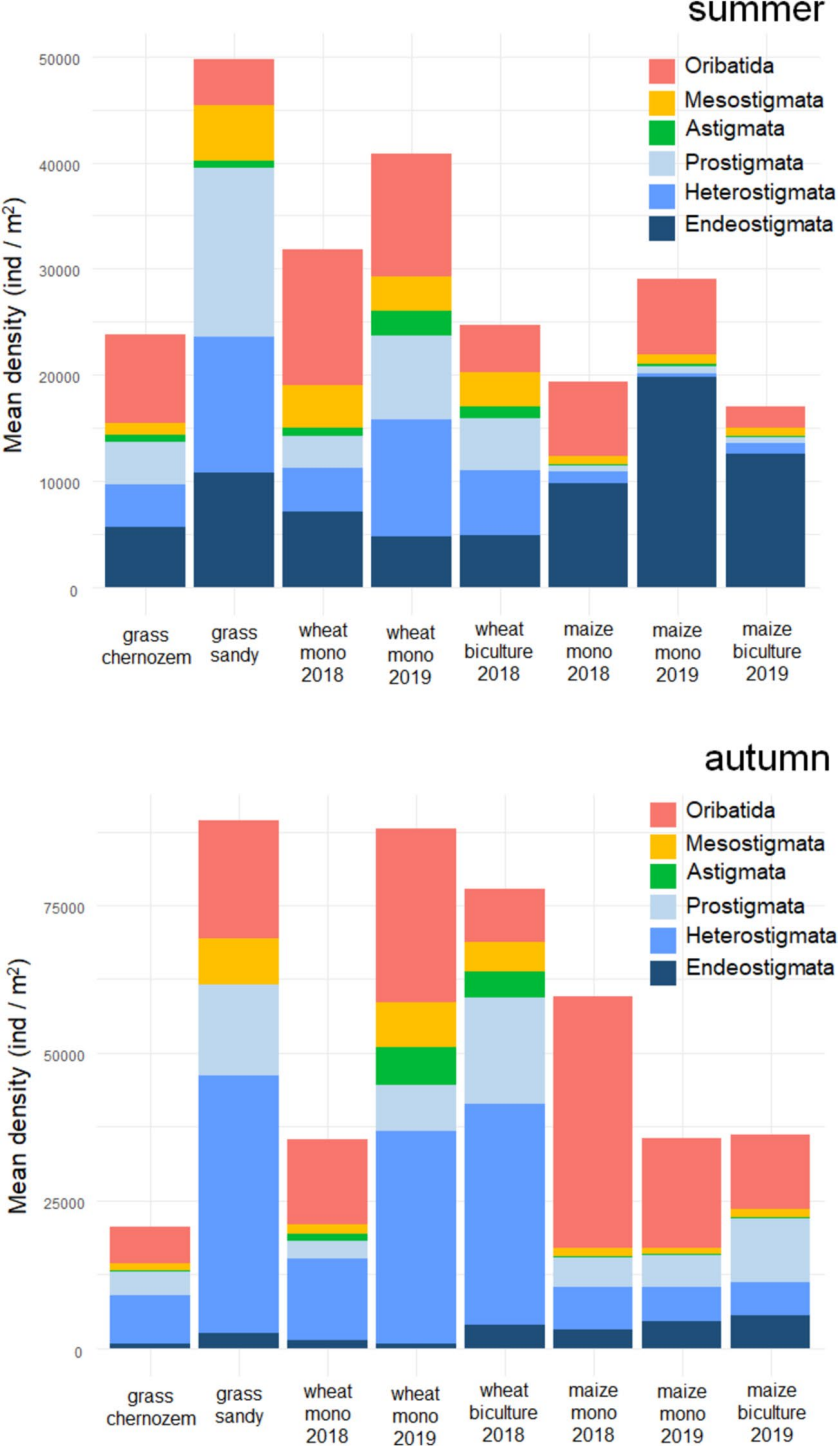
Mean densities of mite groups in the study sites by the seasons

Actinedida (Endeostigmata, Heterostigmata, other Prostigmata) accounted for a significant proportion of the mite assemblages in most of the study sites in all seasons (Fig. 2). Their proportions reached on average 59.0 (± 20.7 SD) % of the total mite number among all the samples, which was similar in two seasons [summer: 64.3 (± 19.1 SD) %, autumn: 53.7 (± 20.9 SD) %]. The total number of soil-dwelling actinedid mites varied between 0 and 29 458 ind. m^−2^ in the summer and between 199 and 95 939 ind. m^−2^ in the autumn.

**Fig. 3:**
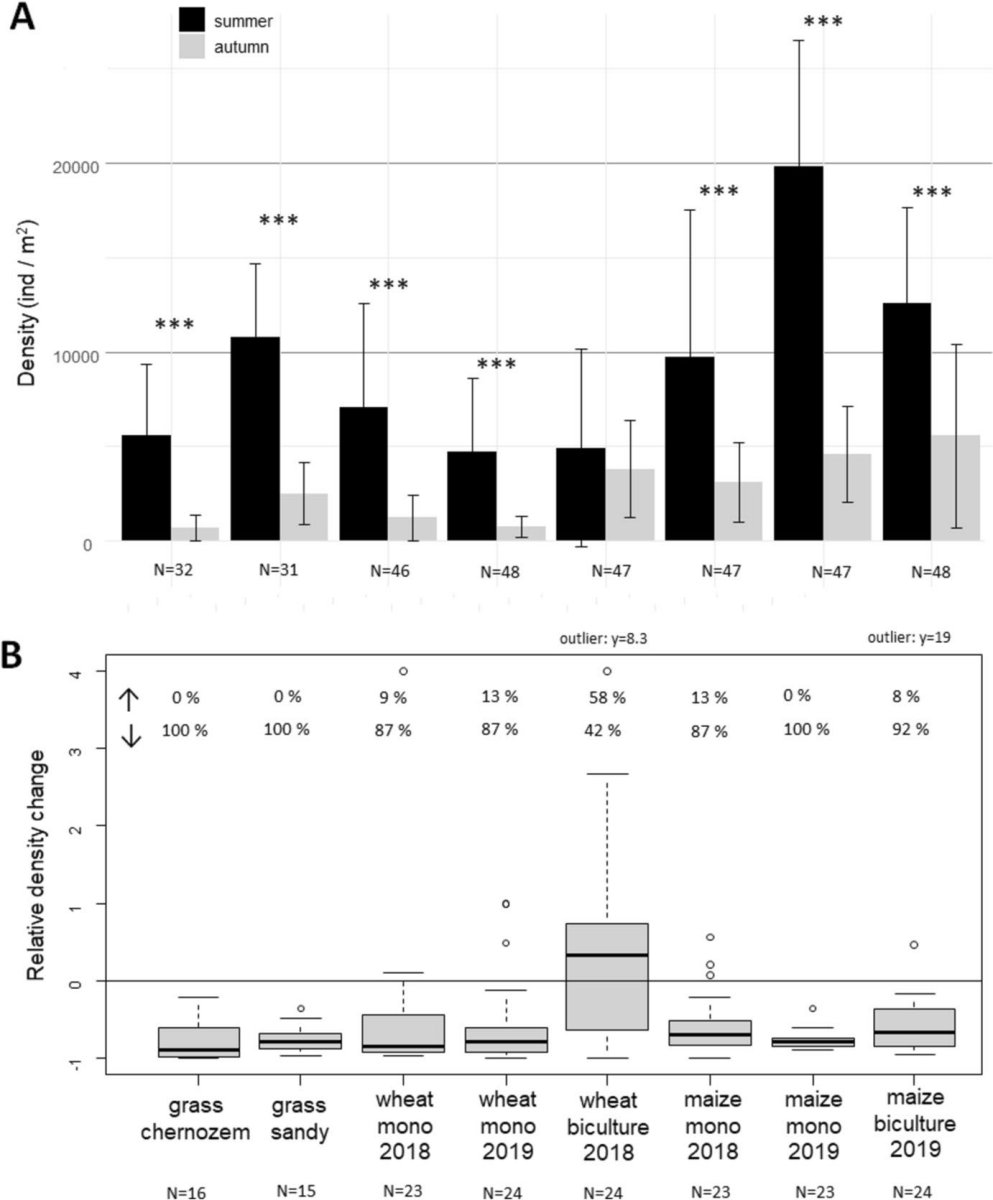
**A**: Densities of endeostigmatid mites in the study sites in the seasons of the same year (mean ± standard error). Significant differences between the two seasons in lm or gls models are shown with asterisks, *p < 0.05, **p < 0.01, ***p < 0.001. **B**: Relative density changes between summer and autumn in the same year. The study sites included three types of plants: winter wheat, summer maize, and a mixture of different grass species. Grass was planted in two types of soil: chernozem and sandy soil. Mono = monoculture, N = number of data points in each category. Percent values indicate the rate of sampling plots, where increase ↑ and decrease ↓ of abundance values was detected between the two seasons

For Endeostigmata, the summer mean densities (4 744–19 797 ind. m^−2^) were always higher than autumn densities (754–5 564 ind. m^−2^) but in the case of wheat biculture (2018), there was not a significant difference (Fig. 3A; Table 2). The relative density changes showed that the density of endeostigmatid mites almost always decreased from the summer to the autumn (Fig. 3B). Relative density changes showed some outliers that belonged to those data where the densities were very low in all seasons. The most different site was the biculture wheat in 2018 where the density of endeostigmatid mites increased by autumn in most samples (Fig. 3B).

**Table 2:** Seasonal effects on log transformed densities of the three mite groups in the different study sites

		Endeostigmata		Heterostigmata		Prostigmata	
Plants	Study sites:	df/t.ratio	p	df/t.ratio*	p	df/t.ratio/F*	p
Grass	Chernozem	26.1/−6.57	**< 0.0001**	30/1.29	0.208	22.4/−1.45	0.162
Grass	Sandy	22.3/−7.89	**< 0.0001**	29/4.77	**< 0.0001**	26.9/−0.57	0.571
Wheat	Mono 2018	117/−5.55	**< 0.0001**	116/5.01	**< 0.0001**	124/−0.75	0.452
Wheat	Mono 2019	117/−4.47	**< 0.0001**	116/5.67	**< 0.0001**	124/−0.50	0.619
Wheat	Rotation 2018	119/0.198	8.44E-01	117/7.78	**< 0.0001**	122/4.81	**< 0.0001**
Maize	Mono 2018	134/−5.12	**< 0.0001**	123/7.0	**< 0.0001**	136/64.8*	**< 0.0001**
Maize	Mono 2019	134/−6.91	**< 0.0001**	126/9.9	**< 0.0001**	136/49.0*	**< 0.0001**
Maize	Rotation 2019	134/−4.64	**< 0.0001**	124/9.0	**< 0.0001**	136/117*	**< 0.0001**

Significant results are marked with bold numbers

Models: log (Acari density) ~ season * site, separately for the three plant species

Significant differences were calculated between summer and autumn within each gls or lm model. Results of Lm model is marked with*

**Fig. 4:**
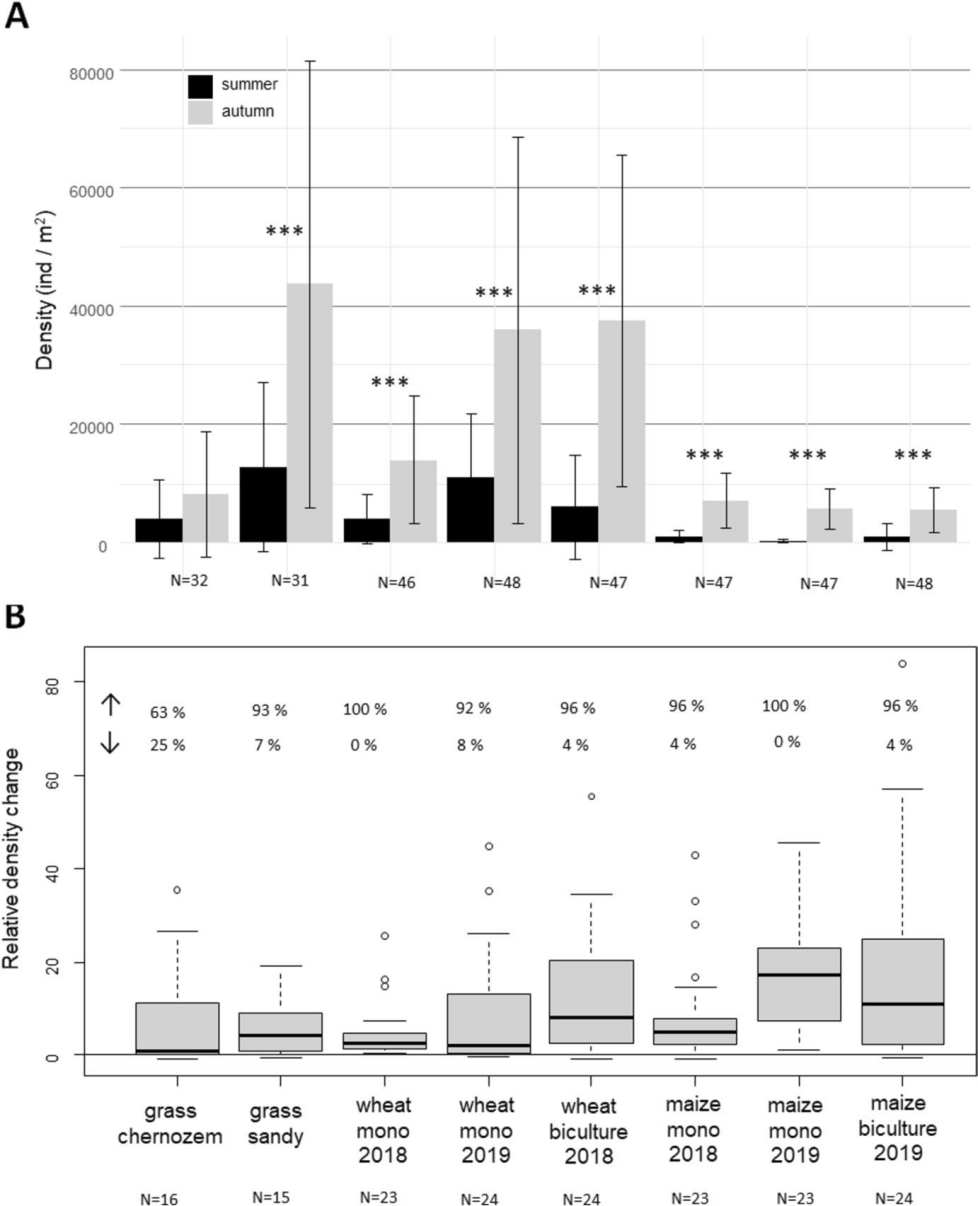
**A**: Densities of heterostigmatid mites in the study sites in the seasons from the same year (mean ± standard error). Significant differences between the two seasons in lm and gls models are shown with asterisks, *p < 0.05, **p < 0.01, ***p < 0.001. **B**: Relative density changes between summer and autumn in the same year. The study sites included three types of plants: winter wheat, summer maize, and a mixture of different grass species. Grass was planted in two types of soil: chernozem and sandy soil. Mono = monoculture, N = number of data points in each category. Percent values indicate the rate of sampling plots, where increase ↑ and decrease ↓ of abundance values was detected between the two seasons

For Heterostigmata, the mean densities were always higher in autumn (5 647–43 653 ind. m^−2^) than in summer (356–12 752 ind. m^−2^, Fig. 4A). The standard deviations of densities are very high in each study site. The total number of heterostigmatid mites almost always increased from summer to the autumn (Fig. 4B). There were only 11 occasions (6% of the total) where the number of heterostigmatid mites decreased from the summer to the autumn. The rate of increase ranged between 0.02 and 84 times increases.

For other Prostigmata, the patterns of seasonal changes depended on site categories. The highest densities were in grass plots of sandy soil (15 680 ind. m^−2^). In maize sites, the total number of prostigmatid mites increased from the summer to the autumn (Fig. S1). In other sites, there were no consistent changes between the two seasons (Fig. S1).

The two different studies measured similar soil parameters and some of them had linear relation with mite densities. For endeostigmatid mites, negative relationships were found between mite densities and soil nitrogen variables (total nitrogen content, soil ammonium and nitrate concentration) in all the study sites; however, not always with the same type of variables (Table 3). In two study sites, Endeostigmata had positive relationship with soil moisture. In sandy soil, soil moisture and total nitrogen content was highly negatively correlated (*r*= − 0.92, *p* < 0.0001), therefore, these two variables were taken into two separate models. For heterostigmatid mites, densities had negative relationships with soil moisture in maize sites and in sandy soil grassland but they had positive relationship with soil moisture in wheat field (Table 3). They had no significant relationships with any soil parameters in chernozem soil.

**Table 3:** Models of Endeostigmata and Heterostigmata mites with the significant soil variables

Response variable	Model	Fixed term					Random term
Log(Endeostigmata density)		Intercept	Explanatory variable(s)				Variance structure
site				F	p	df	
Wheat	lm	2.82	−0.04 × NO_3_	12	0.0009	68	-
Maize	gls	1.13	−0.03 × NH_4_	18.2	0.0001	68	Factor: site category
			0.14 × Moisture	83.6	< 0.0001		
Sand	gls	1.98	0.14 × Moisture	46.2	< 0.0001	28	Factor: season
	gls	11.43	−68.3 × N content	33.1	< 0.0001	28	Factor: season
Chernozem	lm	22.8	−98.9 × N content	15.8	< 0.0001	30	-
Log(Heterostigmata density)				F	p	df	
Wheat	gls	1.31	0.15 × Moisture	12.1	0.0009	67	Factor: site category
Maize	gls	7.34	−0.26 × Moisture	88.8	< 0.0001	69	Moisture
Sand	lm	5.5	−0.13 × Moisture	25	< 0.0001	29	-
Chernozem	n.s.						

*lm* linear regression model, *gls *generalised least squares model

## Discussion

We investigated the seasonal changes of Actinedida from two independent studies. These studies had different plant species, soil types, and management measures from different years making the study conditions very variable. Despite these highly variable backgrounds, we found homogeneous seasonal patterns in actinedid mite densities.

The mean proportion of Actinedid mites in Acari assemblages reached 59% and was similar between summer and autumn. This dominant (> 50%) proportion of Actinedida also occurred in other temperate agricultural fields (Emmanuel et al. 1985; Osler et al. 2008; Van Leeuwen et al. 2015), in tropical crop fields (Kethley 1990) and in grasslands (Norton and Sillman 1985; Kethley 1990; Swarts 1998; Bedano et al. 2006). The high density of prostigmatid mites in agroecosystems might be related to their high stress tolerance in difficult circumstances (Crossley et al. 1992; Russell et al. 2010) such as in low soil organic material (Hutson 1980). However, lower proportion of Prostigmata in agricultural fields are also found in other crop fields (Cao et al. 2011; Yang et al. 2013; Twardowski et al. 2017). Differences between density values of these crop plants may occur due to the intensity of agricultural management (Twardowski et al. 2017), the yearly effect of climate (Osler et al. 2008; Twardowski et al. 2017) or crop plant species (Gruss et al. 2013). However, in some types of agricultural fields they can reach high abundances and as fungivorous microarthropods, they may have an important top-down effect on the ecology soil microbiota.

Seasonality of total number of Prostigmata occur rarely (Gruss et al. 2013; Pacek et al. 2020), however, interesting seasonal patterns of some actinedid mite group appeared in our studies. Endeostigmatid mites had larger populations in summer than in autumn. This pattern might be related to the abiotic, climatic parameters or/and to the biotic, plant physiological and microbiological parameters. The summer was moister and warmer in all study sites and endeostigmatid mites showed positive relationship with soil moisture. In deserts and other dryer habitat types, Endeostigmata populations were found to be related to soil moisture and precipitation (Steinberger and Ben-Ythak 1990; Brantley and Shepherd 2004). In addition, other studies revealed relationships between this mite group and soil organic material as this mite groups occurred mainly on sites with lower organic content (Russell et al. 2010). Endeostigmatid mites also showed negative relations with soil nitrogen content in all of our study sites. In the maize/wheat crop field, we also found that nitrogen fertilisation can be harmful for this mite group (Gergócs et al. 2022a), however, this nitrogen sensitivity is not directly related to seasonal changes.

Most of the soil-dwelling microorganisms in agricultural fields have the same pattern to endeostigmatid mites having an abundance peak during flowering period of crop plant in the summer (Spedding et al. 2004; Ryan et al. 2009; Dong et al. 2014; Bei et al. 2021; Kravchenko et al. 2022). The most common family of Endeostigmata in agriculture fields is Nanorchestidae (Osler et al. 2008; Van Leeuwen et al. 2015). Species in this family are mainly microphytophagous and saprotroph species (Kinnear and Tongway 2004; Gormsen et al. 2006; Russell et al. 2010). This similar pattern may reflect the relationship between soil microorganisms and endeostigmatid mites. These mites may have higher populations because of the higher food supply based on microbiota. It would be important to reveal this relationship in the future as the higher abundance of Endeostigmata may indicate an important characteristic of soil microbiota.

The population increase of the heterostigmatid mite group from summer to autumn was typical for most of our site categories. In other temperate agricultural fields, the heterostigmatid mites showed similar seasonal changes (Emmanuel et al. 1985; Jagger et al. 1988; Cao et al. 2011). The revealed seasonal change with similar density values were also typical in some temperate grasslands (Loots and Ryke 1966; Martin 1978; Clapperton et al. 2002; Neese 2004).

Heterostigmatid mites showed some positive and negative correlations with soil moisture but other soil parameters were not correlated with these mites. According to Steinberger and Whitford (1985) and Bedano et al. (2006), heterostigmatid mites are mainly influenced by soil moisture and temperature. We found that summers were wetter and warmer than autumns in our study sites but there was no consistent negative correlation between soil moisture and mite densities. The increase of heterostigmatid mite abundance might be due to the growth of their populations during the vegetation period which might be interrupted in each year with the negatively influencing cold winter or agricultural management (Martin 1978; Yansheng et al. 2020). Many of the heterostigmatid mites have the special reproduction mode of physogastry (Krantz and Walter 2009) which may make it possible to effectively increase their number in autumn.

During the vegetation period, the plant cover develops and may later die, leaving nutrients and organic matter in the soil which might be useful for heterostigmatid mites (Noble et al. 1996; Clapperton et al. 2002). Heterostigmatid mites in crop lands mainly belong to the families Tarsonemidae, Pygmeophoridae and Scutacaridae (Emmanuel et al. 1985; Crossley et al. 1992; Osler et al. 2008; Yansheng et al. 2020). Within these mite groups, most of the species are fungal feeding microphytophagous or phytphagous species (Gormsen et al. 2006; Krantz and Walter 2009; Yansheng et al. 2020). The changes in their populations might be connected with changes in microbial communities in the soil (Emmanuel et al. 1985; Clapperton et al. 2002; Eitminavičiūtė et al. 2008; Yansheng et al. 2020). In many cases, microbial abundance has a peak during plant development (in the summer), not in the autumn (Kravchenko et al. 2022), but in the autumn, the composition of soil microbiota can be others than in the summer (Bevivino et al. 2014). Therefore, there might be also important changes in soil microbiota which cause these changes in heterostigmatig mites. Thus, in the future, it may be worth to investigate the changes in Heterostigmatid mites that may mean special changes in microbiota in agricultural fields.

Prostigmata (without Heterostigmata) is a various group with many life strategies and feeding habits. Their population changes were more variable than those of the other two mite groups. Their abundance did not change in wheat and in grasslands but increased in maize fields. Other studies also reflected this variability (Loots and Ryke 1966; Jagger et al. 1988). Whenever there would be a pattern, it would be more challenging to explain the reasons as this group is even more various than heteorstigmatid or endeostigmatid mites. Therefore, such a taxonomical level like Prostigmata is insufficient for revealing general ecological patterns.

## Conclusions

We found consistent seasonal soil mite patterns in independent agricultural fields of different locations, soil types, years and managements. Total number of endeostigmatid mites had peaks in summer and heterostigmatid mites had population peaks in autumn. Despite their high abundance, studies about actinedid mite assemblages are under-represented in the agricultural fields. Their seasonal changes in crop lands hide exciting patterns that may help us revealing the critical parameters influencing these mite assemblages and uncover the connections between the development of crop plants, soil-microorganisms and these abundant mite groups. Therefore, these soil-dwelling mites deserve more attention in agricultural soil science even at species level. We assume that Actinedida, mainly Endeostigmata and Heterostigmata are worth to investigate in agricultural fields as a starting point to reveal the connection between the seasonality of soil mites and soil microbiota.

## Electronic supplementary material

Below is the link to the electronic supplementary material.

Supplementary Material 1 (DOCX 60KB)

## Data Availability

The research data are available in the following link: https://zenodo.org/records/14163704. DOI: 10.5281/zenodo.14163703
