# Supplementary material for: Is it worth paying attention to actinedid mites in agricultural fields?
Source: Exp Appl Acarol. 2025 Jan 16;94(2):26. doi: 10.1007/s10493-024-00981-w (PMC11739278; doi:10.1007/s10493-024-00981-w)
Supplement: Supplementary file 1 — Supplementary Material 1 (DOCX 60KB) [file 10493_2024_981_MOESM1_ESM.docx]

**Supplementary materials**


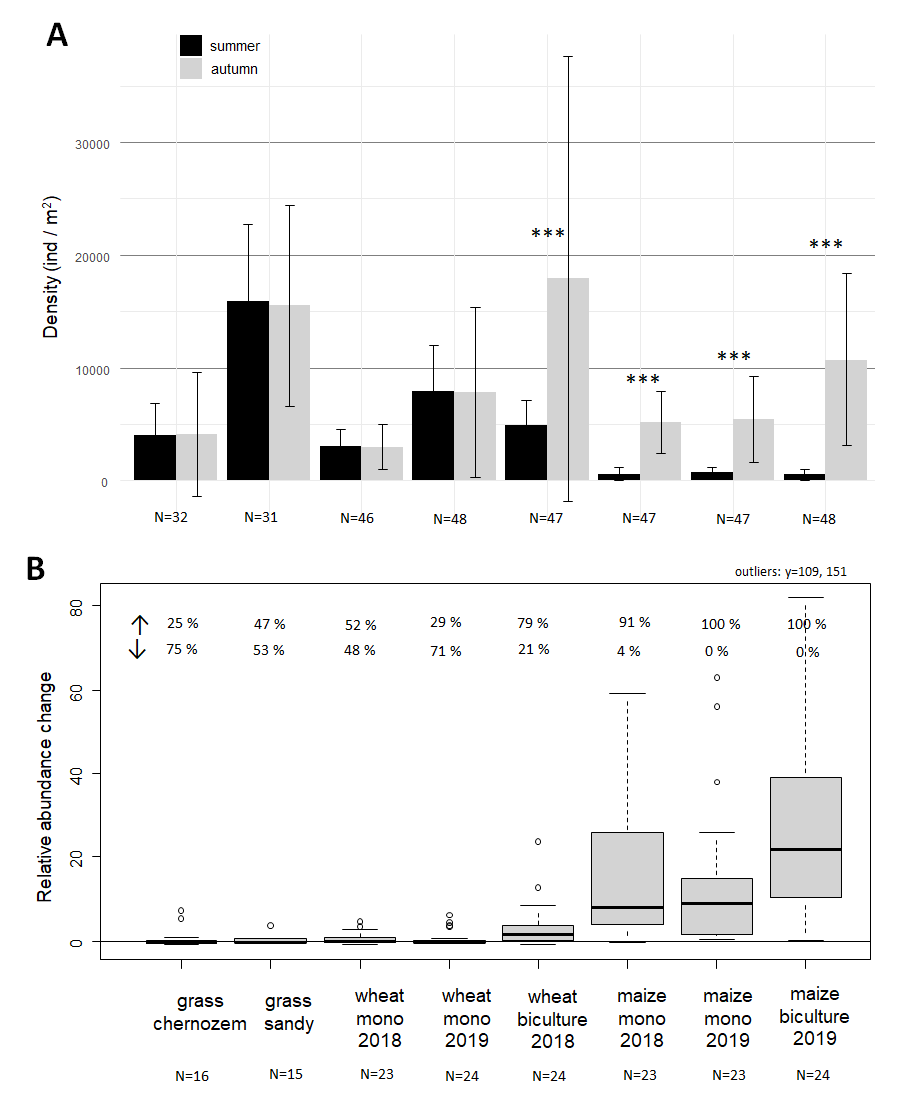


Fig. S1. **A**: Densities of prostigmatid mites in the study sites in the seasons from the same year (mean ± standard error). Significant differences between the two seasons in lm and gls models are shown with asterisks, *p < 0.05, **p < 0.01; ***p < 0.001. **B**: Relative density changes between summer and autumn in the same year. The study sites included three types of plants: winter wheat, summer maize, and a mixture of different grass species. Grass was planted in two types of soil: chernozem and sandy soil. Mono= monoculture, N = number of data points in each category. Percent values indicate the rate of sampling plots, where increase ↑ and decrease ↓ of abundance values was detected between the two seasons.
